# Medial plica is associated with progression of medial meniscus extrusion in middle-aged persons with Kellgren–Lawrence grade 0: data from the osteoarthritis initiative

**DOI:** 10.1186/s13018-025-06472-x

**Published:** 2025-11-24

**Authors:** Shinnosuke Hada, Haruka Kaneko, Takako Aoki, Shuko Nojiri, Martin Englund, Muneaki Ishijima

**Affiliations:** 1 Department of Orthopaedics, Juntendo University Faculty of Medicine, 2-1-1, Hongo, Bunkyo-ku, Tokyo, 113-8421 Japan; 2 Department of Orthopaedic Surgery, Juntendo Tokyo Koto Geriatric Medical Center, Tokyo, Japan; 3 Department of Medicine for Orthopaedics and Motor Organ, Juntendo University Graduate School of Medicine, Tokyo, Japan; 4 Sportology Center, Juntendo University Graduate School of Medicine, Tokyo, Japan; 5 Medical Technology Innovation Center, Juntendo University, Tokyo, Japan; 6 Clinical Epidemiology Unit, Department of Clinical Sciences Lund, Orthopaedics, Faculty of Medicine, Lund University, Lund, Sweden; 7 Department of Community Medicine and Research for Bone and Joint Diseases, Juntendo University Graduate School of Medicine, Tokyo, Japan

**Keywords:** Meniscus, Meniscus extrusion, Osteophytes, Medial plica, Knee osteoarthritis, Magnetic resonance imaging

## Abstract

**Background:**

Medial plica is a structure with limited understanding of its pathology, but it may be related to the development of medial knee osteoarthritis (OA). We examined the association between medial plica and medial meniscus extrusion (MME), progression of MME, and the risk of early-stage knee OA development in middle-aged persons.

**Methods:**

A total of 340 subjects with tibiofemoral Kellgren–Lawrence (K/L) grade 0 from the Osteoarthritis Initiative were included. We evaluated magnetic resonance imaging (MRI) data (right knee) at baseline and six years later. At baseline, the severity of medial plica (grade 0–3), extent of MME (grade 0–3) as well as measured MME in mm was determined. After six years, changes in MME (ΔMME), cartilage loss, and the formation of osteophytes were evaluated.

**Results:**

Medial plica (grade ≥ 1) was observed in 280 subjects (82.4%) at baseline, of whom 13 subjects had grade 3 plica. The MME was 1.7 ± 0.8 mm at baseline and 2.1 ± 0.9 mm after 6 years. Progression of MME was observed in 83 subjects (24.5%) after 6 years. The cartilage loss after 6 years in subjects with medial plica grade ≥ 1 at baseline was greater than that in subjects without medial plica (Δcartilage score 2.9 ± 2.8 vs. 2.2 ± 3.0, *p* = 0.01), although the difference was borderline when analyzed across all plica grades (*p* = 0.05). The ΔMME was greater for higher grades of medial plica at baseline (*p* = 0.01). Subjects with medial plica grade ≥ 1 had a higher risk of progression of MME grade (odds ratio [OR] 2.1, 95% CI 1.0–4.5, *p* = 0.04), but this was not significant for grade ≥ 2 in sensitivity analysis (OR 1.2, *p* = 0.50). Progression of K/L grade was observed only for medial plica grade ≥ 2 (OR 2.1, 95% CI 1.1–4.2, *p* = 0.03), but not for grade ≥ 1.

**Conclusion:**

In middle-aged persons with K/L grade 0, the presence of medial plica—particularly higher-grade lesions—was associated with progression of MME and cartilage loss, and grade ≥ 2 lesions were additionally associated with radiographic progression of knee OA.

## Background

Knee osteoarthritis (OA) is a disease characterized by the degeneration and deformation of the knee joint, such as the articular cartilage, subchondral bone, and meniscus [1]. This may lead to knee symptoms, decreased activity of daily living and quality of life [2]. The number of knee OA patients is steadily increasing with increased longevity and increased levels of obesity and there is no cure [3]. In recent years, attention has been focused on the early pathophysiology of knee OA, with the ultimate aim of changing from symptomatic treatments to disease-modifying strategies aimed at preventing the onset of OA, or to slow, halt, or reverse its progression. To date, the typical imaging diagnosis of knee OA has been by use of plain radiography, and in epidemiologic and clinical studies a grade ≥ 2 according to the Kellgren–Lawrence (K/L) classification is typically defined as radiographic knee OA [4]. However, since "radiographic OA" may represent an already relatively advanced stage, magnetic resonance imaging (MRI) offers opportunities to provide new insights into the pathogenesis of OA at an earlier stage, and may thus identify new opportunities for therapeutic interventions [5, 6].

**Fig. 1:**
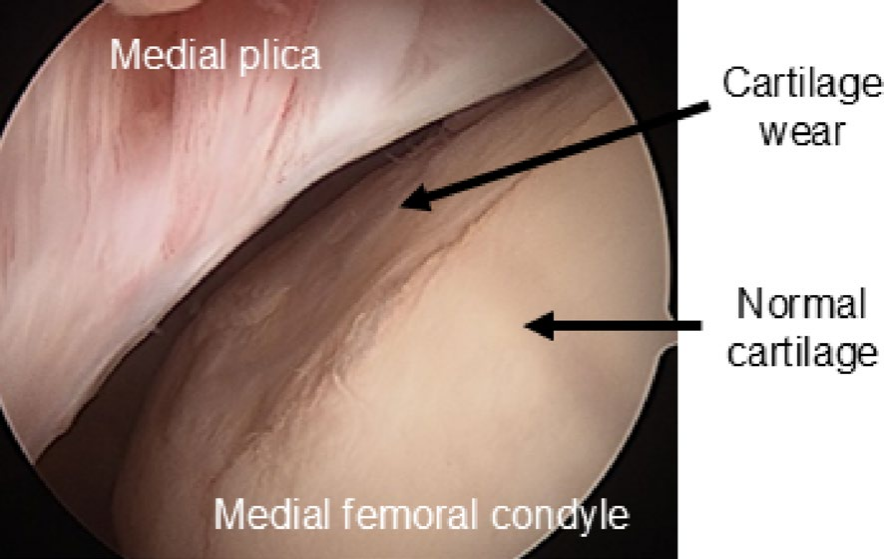
Arthroscopic images of the knee joint in patients with medial abrasion syndrome

Particularly noteworthy is medial meniscus extrusion (MME), which occurs when the medial meniscus extrudes towards the outside of the joint. Such extrusion may be due to meniscus tissue/capsule degeneration, meniscus tears, bone shape changes and osteophyte formation, however, there are still many unclarities about its etiology and pathogenesis [7]. The deviation from the meniscus original position may increase cartilage contact stress, and lead to increased risk of development of both cartilage damage and bone marrow lesions. Thus, MME has been reported to be risk factor for both the onset and progression of knee OA [8–10]. Furthermore, a previous cross-sectional study suggested that cartilage degradation tends to occur more frequently on the medial femoral condyle [11]. This area is often in contact with the medial plica. The medial plica is one of the five types of plicae and originates from remnants of the embryonic septum. Repetitive overuse or trauma can lead to thickening and fibrosis of the plica, which may cause impingement against the medial femoral condyle, resulting in pain, joint effusion, and a popping sensation. A pathological cascade involving synovitis, fibrosis, and reduced elasticity has been proposed to explain this process. [12, 13]. Medial abrasion syndrome, in which the medial plica may rub against the cartilage of the medial femoral condyle, has been reported in 88% of knee OA patients and has been associated with medial synovitis, cartilage degradation, and osteophyte formation [14]. It may thus play an important role in the early pathogenesis of knee OA (Fig. 1).

The pathology of knee OA is however complex and involves various interrelated structures and conditions. Therefore, it is important to gain new insights into these relationships. While there are numerous reports on the relationship between MME and cartilage damage, osteophytes, and subchondral bone, there are no reports on the potential association between medial plica and MME.

Thus, our aim was to determine the association between medial plica and MME and its progression. In addition, we investigated how medial plica and features indicative of development of early-stage knee OA, such as cartilage damage and osteophyte formation, were associated over the six-year follow-up period.

## Materials and methods

### Subjects

We used data from the Osteoarthritis Initiative (OAI) database, which is available for public access at http://www.oai.ucsf.edu/. The OAI is a large ongoing cohort study targeting the characterization of risk factors associated with the onset and progression of symptomatic knee OA and aiming to identify biomarkers of the disease. We hypothesized that medial abrasion syndrome, MME, and osteophyte formation, were early events in OA. Therefore, we selected the youngest subjects in the OAI cohort [15, 16]. Our criteria for sampling were the following:

- Age between 45 and 55 years
- K/L grade of 0 in both knees at baseline, based on central OAI readings
- Knee MRI scans available at baseline, 24, 48, and at 72 months

Participants aged between 45 and 55 years were included to focus on a middle-aged population at risk of early osteoarthritis and to minimize the confounding effects of age-related structural changes. The subjects were drawn from the OAI incidence and progression cohorts as well as the “non-exposed” reference cohort. The subjects in the OAI incidence and progression cohorts all had risk factors for OA, such as being overweight, previous knee trauma and/or surgery, a family history of total knee joint replacement, presence of Heberden's nodes, and repetitive knee bending. The OAI reference cohort had no such risk factors and no pain, aching, or stiffness in either knee in the year prior to the baseline examination. A total of 4,796 participants were included in the OAI data. Among them, 1159 participants 45–55 years old were identified, of whom 557 had a K/L grade of 0. Among these 557 participants, 340 were available for MRI follow-up after 6 years and ultimately became the study sample [15].

The OAI was approved by the respective institutional review boards of the University of California (San Francisco, CA, USA) and the four OAI clinical centers (University of Pittsburgh, Ohio State University, University of Maryland, Baltimore, and Memorial Hospital of Rhode Island). Informed consent was obtained from all of the participants in accordance with the Declaration of Helsinki.

### X ray-based evaluations

We evaluated the K/L grade after 6 years using X-rays and assessed the grade progression from the baseline.

### MRI protocol

Siemens 3 T MRI scanners, one at each of the four clinical study centers located in the United States, were used to obtain baseline (enrollment period 2004–2006) and annual follow-up scans over a period of six years. The imaging protocol included intermediate-weighted turbo spin-echo (IW TSE) MRI scans with the following parameters: repetition time = 3700 ms, echo time = 29 ms, slice thickness = 3 mm, and in-plane resolution of 0.37 × 0.46 mm.

### MRI-based evaluations

For this study, we only report findings from right knees. The observers were blinded to patient information. The extent of MME at baseline and at the 72 months follow-up was measured as the distance from the outermost edge of the medial meniscus to the line connecting the femoral and tibial cortices. MME was classified into 4 subgroups based on the extent of MME: grade 0 (distance < 2.0 mm), grade 1 (2.0–2.9 mm), grade 2 (3.0–4.9 mm), and grade 3 (≥ 5.0 mm). Grade ≥ 1 was classified as positive for MME [7].

**Fig. 2:**
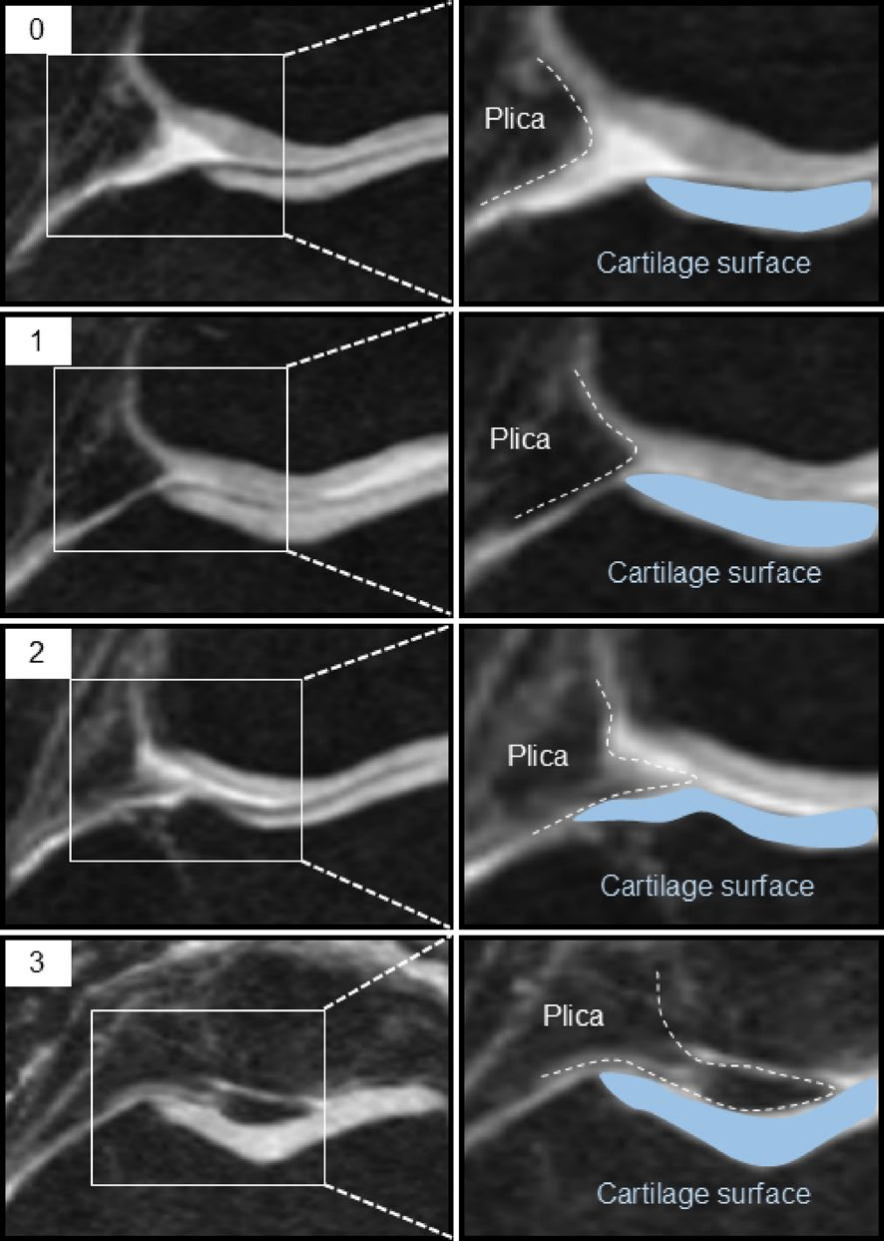
Severity of medial plica on MRI. Grade 0, no obvious plica; grade 1, cord-like elevation in the synovial wall; grade 2, a shelflike appearance but no coverage of the anterior surface of the medial trochlea; grade 3, a large shelf-like appearance and coverage of the anterior surface of the medial trochlea

**Fig. 3:**
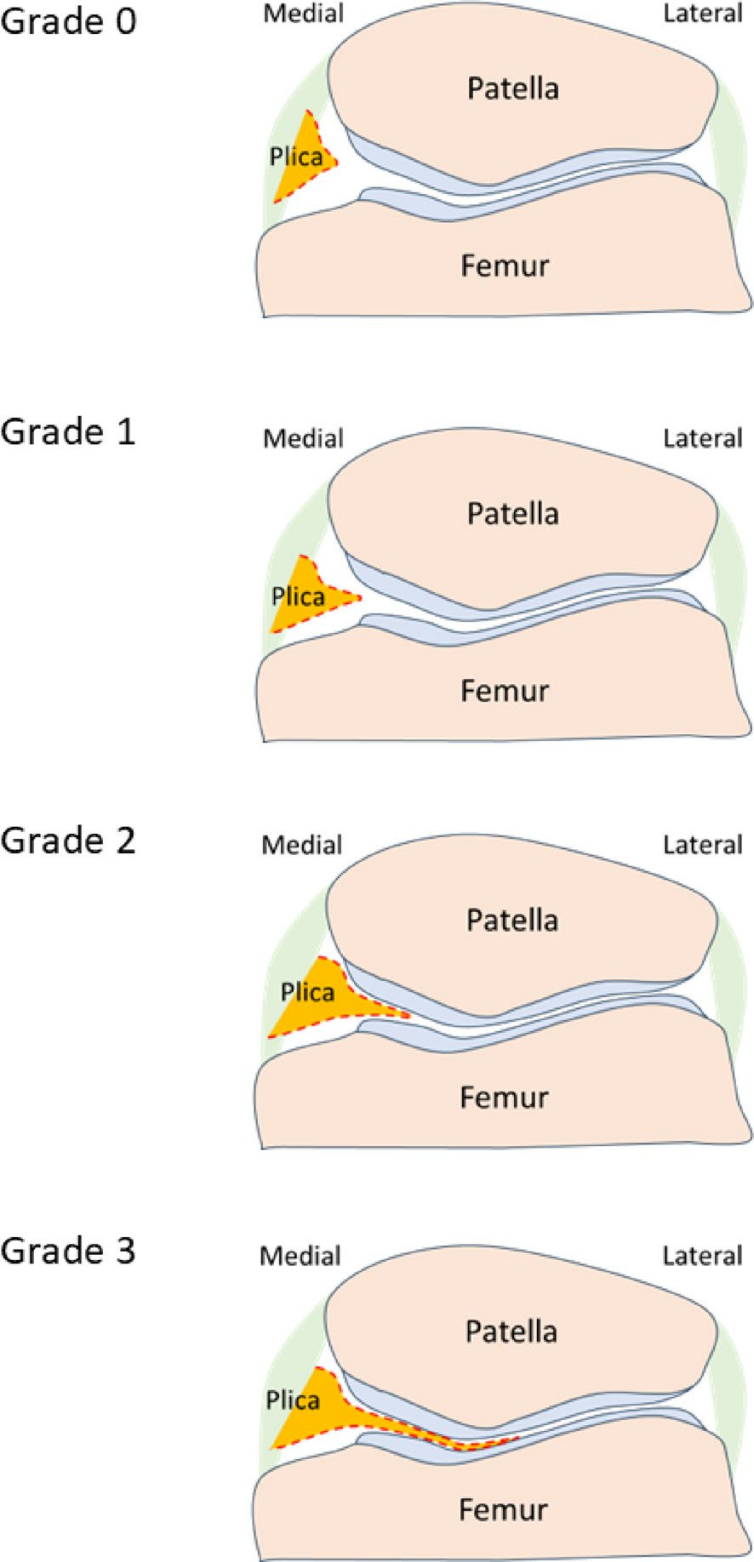
Schematic illustration of medial plica grades corresponding to MRI classification shown in Fig. 2. Grade 0, no discernible plica; grade 1, cord-like elevation in the synovial wall; grade 2, shelf-like configuration without coverage of the anterior surface of the medial trochlea; grade 3, large shelf-like configuration with coverage of the anterior surface of the medial trochlea. This diagram is intended to visually clarify the anatomical location and morphological distinctions of each grade

Medial plica at baseline was graded from 0 to 3 using axial images, according to their size: grade 0 (no obvious plica), grade 1 (cord-like elevation in the synovial wall), grade 2 (small shelf-like appearance but not covering the anterior surface of the medial trochlea), and grade 3 (large shelf-like appearance and covering the anterior surface of the medial trochlea) (Fig. 2, 3) [17].

We classified grade ≥ 1 as positive for medial plica, and we also performed a sensitivity-analysis for ≥ grade 2 as positive.

The presence of MRI features indicative of OA, including cartilage lesions and osteophytes, were scored on baseline and 72-months follow-up MRIs according to the Whole-organ Magnetic Resonance Imaging Score (WORMS) system [18]. Each region of the compartment surface received its own grade according to the previously reported methods [11]. The cartilage morphology was graded from 0 to 6, while osteophytes were graded from 0 to 7. The grades from each subregion were summed for each feature, respectively.

The interclass correlation coefficient (ICC) for interreader agreement was 0.94 (95% confidence interval [CI] 0.91–0.96) for MME, 0.93 (95% CI 0.84–0.96) for medial plica, 0.91 (95% CI 0.66–0.98) for WORMS cartilage score and 0.94 (95% CI 0.91–0.96) for WORMS osteophyte score. The interobserver reproducibility was also high: ICC 0.93 (95% CI 0.76–0.98) for MME, 0.93 (95% CI 0.83–0.95) for medial plica, 0.92 (95% CI 0.67–0.97) for WORMS cartilage score and 0.92 (95% CI 0.86–0.95) for WORMS osteophyte score. Interreader reliability was assessed using a two-way mixed-effects model for single measurements (ICC [1, 3]), based on independent evaluations by two fixed raters (KS and SH). Intrareader reliability was evaluated using the same model, with one rater reassessing the same images after a 4-week washout period to minimize recall bias.

### Statistical analyses

Baseline characteristics were presented as the mean with its standard deviation (SD) or percentage (%), as appropriate. The extent of MME, cartilage score, and osteophyte score were calculated. The associations between medial plica at baseline and change of MME (ΔMME), cartilage loss (Δcartilage score), and osteophyte formation (Δosteophyte score) were examined using linear regression with adjustment for age, sex, and body mass index (BMI). Relative risk (RR) for MME, cartilage loss, osteophyte formation and K/L grade progression if having medial plica were examined using logistic regression with adjustment for age, sex, and BMI.

All tests were two-sided, and statistical significance was set at *p* < 0.05. Statistical analyses were performed using the SPSS software program (version 21.0; SPSS Institute, Chicago, IL, USA).

## Results

One hundred sixty-seven subjects among the 340 subjects (49.1%) were men, and 173 (50.9%) were women. The age of the study subjects was 50.4 (SD: 3.0) years of age. On baseline knee MRI, while no medial plica was observed in 60 subjects (17.6%), medial plica grade was observed in 280 subjects (82.4%). The severity of the plica was grade 1 in 198 (70.7%), grade 2 in 69 (24.6%), and grade 3 in 13 (4.7%) among the subjects with medial plica. At baseline, MME was classified as grade 0 in 236 knees (69.4%), grade 1 in 89 knees (26.2%), grade 2 in 14 knees (4.2%), and grade 3 in 1 knee (0.3%). MME was observed in 104 (30.6%) of the subjects and it was 1.7 (0.8) mm on average. The severity of MME was grade 1 in 89 (85.6%), grade 2 in 14 (13.4%), and grade 3 in 1 subject (1.0%) among the subjects with MME (Table 1).

**Table 1:** Baseline characteristics of the subjects with Kellgren–Lawrence grade 0

Characteristic	n = 340
Sex (male/female), n (%)	167 (49.1)/173 (50.9)
Age, mean (SD), years	50.4 (3.0)
BMI; mean (SD), kg/cm^2^	26.7 (4.4)
*Medial plica, n (%)*	
Grade 0	60 (17.6)
Grade 1	198 (58.2)
Grade 2	69 (20.3)
Grade 3	13 (3.8)
*MME, n (%)*	
Grade 0	236 (69.4)
Grade 1	89 (26.2)
Grade 2	14 (4.1)
Grade 3	1 (0.003)
MME mean (SD), mm	1.7 (0.8)
*WORMS*	
Osteophyte score (0–35), mean (SD)	10.1 (6.2)
Cartilage score (0–30), mean (SD)	2.9 (3.3)

BMI, body mass index; MME, medial meniscus extrusion; WORMS, whole organ magnetic resonance imaging score

At the 6-year follow-up, the distribution changed to 173 knees (50.9%) with grade 0, 121 knees (35.6%) with grade 1, 41 knees (12.1%) with grade 2, and 5 knees (1.5%) with grade 3. The MME had increased at the 6-year follow-up compared to baseline (2.1 [SD: 0.9] mm vs. 1.7 [SD: 0.8] mm) (Table 2).

**Table 2:** MRI-detected structural changes of the medial tibiofemoral compartment of subjects with Kellgren–Lawrence grade 0 at baseline

	Baseline	6-year follow-up	Δ	*p* value
MME, mean (SD) mm	1.7 (0.8)	2.1 (0.9)	0.4 (0.6)	< 0.001
*WORMS, mean (SD)*				
Cartilage (range 0–84)	2.9 (3.3)	5.8 (4.6)	2.8 (2.8)	< 0.001
Medial (range 0–42)	1.7 (2.1)	3.3 (2.8)	1.6 (1.8)	< 0.001
Femur (range 0–18)	0.7 (1.1)	1.5 (1.5)	0.7 (1.1)	< 0.001
Tibia (range 0–18)	0.3 (0.7)	0.6 (1.0)	0.3 (0.7)	< 0.001
Patella (range 0–6)	0.7 (1.1)	1.3 (1.4)	0.6 (0.9)	< 0.001
Osteophyte (range 0–98)	10.1 (6.2)	13.2 (7.6)	3.1 (3.9)	< 0.001
Medial (range 0–49)	5.2 (3.4)	6.8 (4.2)	1.5 (2.4)	< 0.001
Femur (range 0–21)	2.4 (2.0)	3.0 (2.3)	0.6 (1.3)	< 0.001
Tibia (range 0–21)	1.7 (1.5)	2.3 (1.8)	0.6 (1.1)	< 0.001
Patella (range 0–7)	1.1 (1.0)	1.4 (1.1)	0.3 (0.7)	< 0.001

MRI, magnetic resonance imaging; MME, medial meniscus extrusion; WORMS, whole-organ magnetic resonance imaging score

The WORMS cartilage lesion score at 6-year follow-up (5.8 [SD: 4.6]) and osteophyte score (13.2 [SD: 7.6]) were increased compared to baseline (2.9 [SD: 3.3], and 10.1 [SD: 6.2], respectively). There were similar findings for both cartilage lesion and osteophyte score in femur, tibia, and patella, respectively (Table 2).

Group-wise mean changes were first compared using linear regression with plica grade as a categorical variable and adjustment for covariates (Table 2). Subsequently, multivariable models were used to estimate effect sizes and confidence intervals (Table 3). Progression of MME was observed in 83 subjects (24%) after 6 years and the ΔMME was 0.4 (SD: 0.6) mm. The ΔMME increased according to the severity of medial plica at baseline (no plica: 0.2 [SD: 0.5] mm, grade 1: 0.4 [SD: 0.6] mm, grade 2: 0.4 [SD: 0.7] mm, grade 3: 0.9 [SD: 1.5] mm, *p* = 0.002 adjusted by age, sex, and BMI) (Table 3).

**Table 3:** Relationship between medial plica grade at baseline and the development of medial meniscus extrusion (MME), cartilage lesions, and osteophytes over 6 years in the subjects with Kellgren–Lawrence grade 0 at baseline

Plica	ΔMME (mm)	ΔOsteophyte score	ΔCartilage score
–	0.2 (0.5)	3.2 (3.8)	2.2 (3.0)
Grade 1	0.4 (0.6)	2.9 (3.3)	2.9 (2.6)
Grade 2	0.4 (0.7)	2.7 (4.2)	3.2 (3.3)
Grade 3	0.9 (1.5)	6.3 (7.9)	2.5 (2.3)
*p* value (unadjusted)	0.002	0.18	0.05
*p* value (adjusted)	0.002	0.17	0.05

Data are expressed as the mean value (standard deviation). Linear regression analysis (adjustment for age, sex, and body mass index). *P* values were derived from linear regression models treating plica grade as a categorical variable. Effect sizes are not reported in this table; for adjusted estimates, please refer to Table 3

On the other hand, the Δosteophyte score was not associated with medial plica at baseline (*p* = 0.17 adjusted by age, sex, and BMI) (Table 3). Although Δcartilage score tended to increase according to the severity of medial plica at baseline, no essential differences of the Δcartilage score was observed between the subgroups divided by the severity of medial plica at baseline (*p* = 0.05 adjusted by age, sex, and BMI) (Table 3).

Linear regression analysis adjusted for age, sex, and body mass index revealed that, compared to participants with medial plica grade 0, those with grade 2 or higher showed a significantly greater increase in medial meniscus extrusion (ΔMME) over 6 years (β = 0.26 mm, 95% CI 0.06–0.47, *p* = 0.01). Grade 1 showed a non-significant increase in ΔMME (β = 0.15 mm, 95% CI –0.03 to 0.32, *p* = 0.10). No significant associations were observed between medial plica grade and the change in osteophyte score (grade 1: β = –0.40, 95% CI− 1.54 to 0.73, *p* = 0.49; grade 2 + : β = –0.01, 95% CI − 1.31 to 1.29, *p* = 0.99). For cartilage score, both grade 1 (β = 0.74, 95% CI − 0.08 to 1.56, *p* = 0.075) and grade 2 + (β = 0.89, 95% CI − 0.05 to 1.83, *p* = 0.063) showed numerically higher values compared to grade 0, although these differences did not reach statistical significance. (Table 4).

**Table 4:** The association between medial plica grade at baseline in subjects with Kellgren–Lawrence grade 0 and the development of medial meniscal extrusion (MME), osteophytes and cartilage lesions over 6-years; results from linear regression adjusted for age, sex and body mass index

Medial plica grade	Beta coefficient	95% CI	*p* value
*ΔMME* (mm)			
Grade 0	Ref		
Grade 1	0.15	− 0.03–0.32	0.10
Grade 2 +	0.26	0.06–-0.47	0.01
*ΔOsteophyte score*			
Grade 0	Ref		
Grade 1	− 0.40	− 1.54–0.73	0.49
Grade 2 +	− 0.01	− 1.31–1.29	0.99
*ΔCartilage score*			
Grade 0	Ref		
Grade 1	0.74	− 0.08–1.56	0.075
Grade 2 +	0.89	− 0.05–1.83	0.063

CI, confidence interval

The ΔMME of the subjects with medial plica (grade ≥ 1: 0.4 [SD: 0.6] mm) at baseline was significantly greater than that of those without medial plica at baseline (grade < 1: 0.2 [SD: 0.5] mm) (p = 0.03). However, no difference of the Δosteophyte score was observed between subgroups divided by the presence (grade ≥ 1: 3.2 [SD: 3.8] mm) or absence (grade < 1: 2.9 [SD: 3.9] mm) of medial plica at baseline. In contrast, the Δcartilage score in subjects with medial plica (grade ≥ 1: 2.9 [2.8] mm) at baseline was significantly greater than in those without medial plica (grade < 1: 2.2 [3.0] mm) (*p* = 0.01). In the sensitivity analysis where the presence of medial plica was redefined from grade ≥ 1 to grade ≥ 2 at baseline, ΔMME with medial plica (grade ≥ 2: 0.5 [0.8] mm) at baseline was also significantly greater than that in those without medial plica (grade < 2: 0.3 [0.5] mm) (*p* = 0.04). However, no essential differences of either Δosteophyte score and Δcartilage score was observed between subgroups divided by the presence (grade ≥ 2) or absence (grade < 2) of medial plica at baseline.

When the presence of medial plica was defined by grade ≥ 1, the subjects with medial plica had a higher risk of progression of MME grade than those without medial plica (OR 2.1, 95% CI 1.0–4.5, *p* = 0.04; estimated using multivariable logistic regression adjusted for age, sex, and BMI). In the sensitivity analysis (medial plica grade ≥ 2), the point estimate of association was reduced and not statistically significant (OR 1.2, 95% CI 0.7–2.1, *p* = 0.50; logistic regression adjusted for the same covariates) (Table 5). There were 42 subjects whose K/L grade had progressed to ≥ 1 at 6 years (Table 6).

**Table 5:** Risk for progression of medial meniscus extrusion (MME) grade over 6 years by the presence of medial plica in subjects with Kellgren–Lawrence grade 0 at baseline; results from logistic regression adjusted for age, sex and body mass index

Plica grade ≥ 1	Progression of MME grade			Unadjusted RR (95% CI), *p* value	Adjusted RR (95% CI), *p* value
	−	+			
−	51	9	15.0%	2.1 (1.0–4.4), 0.05	2.1(1.0–4.5), 0.04
+	205	75	26.7%		

Plica grade ≥ 2	Progression of MME grade			Unadjusted RR (95% CI), *p* value	Adjusted RR (95% CI), *p* value
	−	+			
−	196	61	23.7%	1.2 (0.7–2.2), 0.47	1.2 (0.7–2.1), 0.50
+	60	23	27.7%		

RR, relative risk; CI, confidence interval

**Table 6:** Knee osteoarthritis severity at the 6-year follow-up of the subjects with Kellgren–Lawrence (K/L) grade 0 at baseline

K/L grade	6-year follow-up
0	298 (87.6%)
1	29 (8.6%)
2	11 (3.2%)
3	2 (0.6%)
4	0 (0%)

While medial plica grade ≥ 1 was not a risk factor for progression of K/L grade (OR 0.9, 95% CI 0.4–2.1, *p* = 0.06; logistic regression adjusted for age, sex, and BMI), medial plica grade ≥ 2 was associated with K/L grade progression compared to grade ≤ 1 (OR 2.1, 95% CI 1.1–4.2, *p* = 0.03; logistic regression adjusted for the same covariates) (Table 7).

**Table 7:** Risk for progression of Kellgren–Lawrence (K/L) grade over 6 years by the presence of medial plica in subjects with K/L grade 0 at baseline; results from logistic regression adjusted for age, sex and body mass index

Plica grade ≥ 1	Progression of K/L grade			Unadjusted RR(95% CI), *p* value	Adjusted RR(95% CI), *p* value
	−	+			
−	52	8	13.3%	0.9 (0.4–2.1), 0.80	0.9 (0.4–2.1), 0.80
+	246	34	12.1%		

Plica grade ≥ 2	Progression of K/L grade			Unadjusted RR(95% CI), *p* value	Adjusted RR(95% CI), *p* value
	−	+			
−	231	26	10.1%	2.1 (1.1–4.2), 0.03	2.1 (1.1–4.2), 0.03
+	67	16	19.2%		

RR, relative risk; CI, confidence interval

## Discussion

This study suggests that, in middle-aged persons with K/L grade 0 knees, the presence of medial plica is a risk factor for the progression of MME, and the higher the grade of the medial plica, the greater the risk of progression of MME. Furthermore, the presence of a high grade of medial plica is associated with radiographic progression of knee OA. In addition, the medial plica was associated with progression of cartilage damage on MRI. For the first time, these results suggest that medial plica may be a structure involved in the early-stage knee OA pathogenesis, and it may potentially be a new treatment target aimed for prevention of knee OA.

**Fig. 4:**
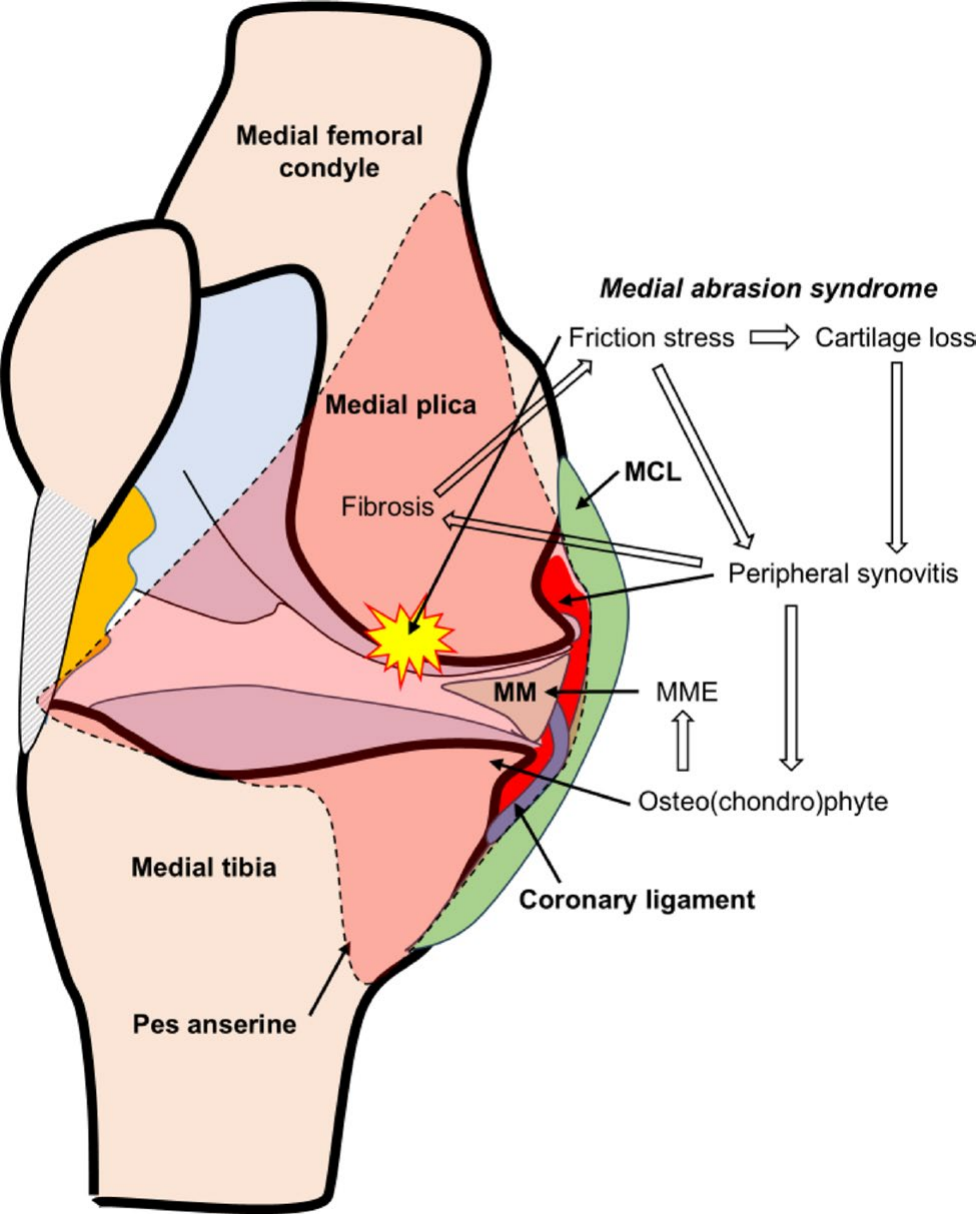
Schematic illustrations depicting our hypothesis of development of MME in subjects with medial plica. Increased friction stress between the medial plica and articular surface of the medial femoral condyle caused by medial abrasion syndrome induces cartilage loss. Cartilage loss induces peripheral synovitis, osteophytes formation, and fibrosis of the medial plica. As the medial plica is connected to the attachment site of the gracilis tendon, namely the pes anserine, fibrosis of the medial plica increases traction stress. Osteophyte formation begins at the peripheral areas of the articular cartilage, and medial meniscus extrusion (MME) may be induced by displacement of the coronary ligament by the tibial osteophyte. Osteophyte-mediated MME may also promote the degradation and destruction of articular cartilage owing to increased mechanical stress on these joint structures, leading to the development of knee OA. MM, medial meniscus. MCL, medial collateral ligament

When the knee joint is flexed more than 50°, the medial plica may generate friction on the cartilage surface of the medial femoral condyle with increased shear force [10], which may stimulate the production of interleukin-1β and the expression of matrix metalloproteinase-3, triggering synovitis [19, 20]. Synovitis may lead to hypertrophy of the synovium at the joint periphery as well as endochondral ossification, leading to the formation osteophytes [21]. We have previously suggested that MME may be induced by osteophytes in early-stage knee OA [22–24]. Therefore, we hypothesize that osteophytes, formed as a result of synovitis triggered by medial abrasion syndrome, could potentially induce MME (Fig. 4). However, whether the medial plica, osteophyte, and medial meniscus truly interact mechanically remains unclear. The dynamic relationship among these structures during knee motion has not been fully elucidated, and further studies are needed to clarify their potential mechanical involvement in disease progression.

Our longitudinal study suggests that medial plica was not only associated with increased MME but also increased risk of cartilage damage. This aligns with a previous cross-sectional study in which the presence of medial plica was associated with cartilage damage in the patellofemoral joint [17]. Medial abrasion syndrome may lead to cartilage degradation centered around the medial condyle of the femur. Concomitant synovitis, as well as the subsequent fibrosis and hypertrophy of the medial plica, may hypothetically further increase the shear stresses on the medial femoral cartilage, which may accelerate cartilage degradation and synovitis, potentially creating a vicious cycle [11, 14]. In addition, the medial plica may undergo qualitative changes over time, such as loss of elasticity and hypertrophy, following inflammatory processes. Previous studies have shown that the more severe the OA changes, the larger the medial plica tends to be [25]. Therefore, in the present study, we focused on K/L grade 0 knees, in which such inflammatory and fibrotic changes are expected to be minimal. However, because the degree of joint effusion and the knee flexion angle at the time of MRI can affect the contact between the plica and the cartilage surface, there remains a possibility of misgrading the plica based on MRI alone. This highlights the need to develop more rigorous evaluation methods in future studies. Moreover, future analyses should address differences in cartilage wear between areas covered and not covered by the plica, as well as pathological assessments of the plica’s qualitative characteristics, rather than focusing solely on its size. In recent years, suture techniques targeting the degenerative meniscus or MME have been explored as a potential treatment for early-stage knee OA, but the results have been disappointing [26]. Still, medial plica may become a target for further early-stage knee OA treatments. For instance, young patients with chronic knee pain and a large medial plica may undergo resection of the plica. However, when established knee OA and medial abrasion syndrome coexist, it has been reported that resection of the medial plica alone is associated with poor results [19]. In patients with knee OA, arthroscopic medial release, in which the medial plica is excised and the tightened medial joint capsule is incised, has been reported to improve joint pain, radiographic joint space width, and varus alignment [27]. However, because this was not a comparative study and was based on radiographic evaluations, it remains unclear whether these improvements were truly attributable to medial plica excision. Further controlled studies with longitudinal MRI assessments are needed to clarify the clinical impact of treating the medial plica. Considering the mechanism underlying synovial thickening from the inflammatory synovial membrane, which differentiates into osteophytes through the process of endochondral ossification [21], progression of OA may hypothetically be prevented or slowed down by targeting synovitis and osteophytes in their early stages.

Although the present study is focused on the medial plica, the relationships with other structures, such as the pes anserine, medial meniscus integrity, and medial collateral ligament should also be examined. Further, as no direct evaluation of synovitis has been performed, the association with synovitis remains unclear.

In conclusion, in middle-aged subjects with K/L grade 0 knees, the presence of medial plica and its severity was found to be associated with progression of MME and K/L grade as well as increased risk of cartilage loss.

## Data Availability

The datasets analyzed during the current study are available in the Osteoarthritis Initiative (OAI) repository: https://nda.nih.gov/oai/.
